# IL33: Roles in Allergic Inflammation and Therapeutic Perspectives

**DOI:** 10.3389/fimmu.2019.00364

**Published:** 2019-03-04

**Authors:** Ben C. L. Chan, Christopher W. K. Lam, Lai-Shan Tam, Chun K. Wong

**Affiliations:** ^1^State Key Laboratory of Research on Bioactivities and Clinical Applications of Medicinal Plants, Institute of Chinese Medicine, The Chinese University of Hong Kong, Shatin, Hong Kong; ^2^State Key Laboratory of Quality Research in Chinese Medicines, Macau Institute for Applied Research in Medicine and Health, Macau University of Science and Technology, Taipa, Macau; ^3^Department of Medicine and Therapeutics, The Chinese University of Hong Kong, Shatin, Hong Kong; ^4^Department of Chemical Pathology, The Chinese University of Hong Kong, Shatin, Hong Kong

**Keywords:** IL-33, allergic inflammation, signal transduction, eosinophils, mast cells, innate lymphoid cells (ILC), Chinese herbal medicine, therapeutics

## Abstract

Interleukin (IL)-33 belongs to IL-1 cytokine family which is constitutively produced from the structural and lining cells including fibroblasts, endothelial cells, and epithelial cells of skin, gastrointestinal tract, and lungs that are exposed to the environment. Different from most cytokines that are actively secreted from cells, nuclear cytokine IL-33 is passively released during cell necrosis or when tissues are damaged, suggesting that it may function as an alarmin that alerts the immune system after endothelial or epithelial cell damage during infection, physical stress, or trauma. IL-33 plays important roles in type-2 innate immunity via activation of allergic inflammation-related eosinophils, basophils, mast cells, macrophages, and group 2 innate lymphoid cells (ILC2s) through its receptor ST2. In this review, we focus on the recent advances of the underlying intercellular and intracellular mechanisms by which IL-33 can regulate the allergic inflammation in various allergic diseases including allergic asthma and atopic dermatitis. The future pharmacological strategy and application of traditional Chinese medicines targeting the IL-33/ST2 axis for anti-inflammatory therapy of allergic diseases were also discussed.

## Introduction

Interleukin33 (IL-33) is a member of the IL-1 cytokine family that includes IL-1α, IL-1β, and IL-18 (1) and constitutively expressed in structural and lining cells including fibroblasts, endothelial, and epithelial cells of skin, gastrointestinal tract, and lungs that are exposed to the environment (2). IL-33 lacks a secretory signal peptide encoded by the *Il1rl1* gene (1), an IL-1 family trait for releasing via the classical endoplasmic reticulum and Golgi pathway (1). Under the inactive state, IL-33 is harbored in the cell nuclei and associated with chromatin by a chromatin-binding motif, belonging to the cellular homeostasis and acting as a transcriptional repressor (2, 3). The N-terminus of IL-33 contains a nuclear localization sequence, a homeodomain-like helix-turn-helix DNA-binding domain and a chromatin-binding domain (3). Different from most cytokines that are actively secreted from cells, IL-33 is released passively in its full length form (amino acids 1–270, IL-33_FL_) during cell necrosis, cellular activation through ATP signaling without cell death or when tissues are damaged, suggesting that it may function as an alarmin that alerts the immune system after endothelial or epithelial cell damage during infection, physical stress or trauma (4, 5). IL-33 activates signaling pathways depending on the myeloid differentiation primary response gene 88 (Myd88) of immune cells expressing the cytokine receptor interleukin 1 receptor-like 1 (ST2) and signals through a heterodimeric receptor complex comprising an IL-33-specific ST2 coupled with the co-receptor IL-1 receptor accessory protein (IL-1 RAcP) (6, 7). ST2 is selectively and stably expressed on the cell surface of Th2 cells (8), CD4+ T cells, group 2 innate lymphoid cells (ILC2s) and also other immune cells such as mast cells, basophils, eosinophils, macrophages, dendritic cells and natural killer cells (9–18). Signaling of IL-33 can be activated through nuclear factor kappa-B (NF-κB), c-Jun N-terminal kinase (JNK), and p38 mitogen activated protein kinase (MAPK) cascades (19).

In humans, both IL-33 mRNA and protein are substantially elevated in the inflamed skin lesions of atopic dermatitis (AD) patients when compared with non-inflamed skin (20). IL-33 is a Th2-oriented cytokine which enhances the production of Th2 cytokines, particularly IL-5 and IL-13 (21). In addition, IL-33 is also a chemoattractant for Th2 cells *in vitro* and *in vivo*, indicating the importance of IL-33 in Th2 cells mobilization (22). In large-scale genome-wide association studies, genes encoding IL-33 and its receptors have been identified as susceptibility loci in asthma (23–26).The alarmin activities of IL-33 are regulated at multiple levels (6, 7, 27). Several hours after its extracellular release, IL-33_FL_ is transiently inactivated by oxidation of critical cysteine residues (28). Inflammatory proteases from immune cells such as neutrophils (cathepsin G and elastase) and mast cells (chymase and tryptase) degrade IL-33_FL_ into shorter mature forms containing the C-terminal IL-1-like cytokine domain with much higher activity than IL-33_FL_ (29). A recent study has further shown that IL-33_FL_ functions as a protease sensor that detects proteolytic activities associated with various environmental allergens including house dust mite, pollens, bacteria and fungi (30). When exposed to allergen proteases, IL-33_FL_ is rapidly cleaved in its central “sensor” domain, which leads to the activation of the generation of ILC2s, and allergic inflammation can be reduced by preventing the IL-33_FL_ cleavage (30, 31). In this review, we focus on the recent advances of the underlying intercellular and intracellular mechanisms by which IL-33 can regulate various key immune cells in the allergic inflammatory diseases including allergic asthma and AD, and the future pharmacological strategy and the potential application of traditional Chinese medicines targeting the IL-33/ST2 axis for the treatment of allergic inflammatory diseases.

## Effects of IL-33 on Immune Cells Activation in Allergic Inflammations

### Eosinophils

IL-33 potently induces eosinophilia in *in vivo* murine models (32, 33) and activates eosinophils, the principal effector cells in allergic inflammation, to produce superoxide (34), upregulates the expression of adhesion molecules and enhances eosinophil survival (35), suggesting that it can play an important role in the exacerbation of inflammation in allergic diseases mediated by the activation of eosinophils. Polymorphism of human IL-33 and ST2 genes has been shown to associate with increased numbers of eosinophils (36). In our previous studies, we have shown the activation of eosinophils, by different stimuli and its interactions with structural cells in atopic dermatitis (AD) and allergic asthma (37–44). Such findings showed that intercellular interaction of eosinophils and dermal fibroblasts could provoke the release of pro-inflammatory cytokines and chemokines, implying the pathogenic effects of eosinophils infiltration in the inner dermal fibroblast layer in AD skin lesions.

In our study of allergic inflammation, IL-33 significantly promote eosinophil survival and cell surface expression of the adhesion molecule intercellular adhesion molecule (ICAM)-1, but ICAM-3, and L-selectin expressions were suppressed. In addition, IL-33 stimulates significant release of pro-inflammatory cytokine IL-6 and the chemokines CXCL8 and CCL2 from eosinophils (41).The release of cytokines and chemokines were differentially regulated by the activation of nuclear factor (NF)-kB, p38 mitogen-activated protein kinase (MAPK) and extracellular signal-regulated kinase (ERK) pathways in eosinophils (41, 45). In our study of IL-33 in AD using eosinophils and fibroblasts co-culture, we found that there was significant increase in the production of pro-inflammatory cytokines such as IL-6 and AD-associated chemokines CXCL1, CXCL10, CCL2, and CCL5 (45). Such increase was further upregulated by IL-33 stimulation, and significant production of CXCL8 from eosinophils and fibroblasts co-culture was observed (42). The main source in co-culture for the release of CCL5, and IL-6, CXCL1, CXCL8, CXCL10, and CCL2 was eosinophils and fibroblasts, respectively, and direct contact between eosinophils and fibroblasts was essential for the release of AD-related chemokine CXCL1, CXCL10, CXCL8, and CCL5. IL-33 stimulation also upregulated the cell surface expression of intercellular adhesion molecule-1 (ICAM-1) on both eosinophils and fibroblasts in co-culture, with differential activation of ERK, JNK, p38 MAPK, NF-kB, and phosphatidylinositol 3-kinase–Akt (PI3K/Akt) pathways (42).

### T Cells and ILC2

Besides eosinophils, IL-33 has also been shown to be an active and soluble co-stimulator of T cells, by promoting the expansion and functional differentiation of both effector T cells and GATA-3+ regulatory T cells (9, 46). Study of IL-33 signaling-deficient mice has also demonstrated the crucial role of IL-33 in protective anti-viral T cell immunity (9). Activated Th1 and CD8+ T cells have been shown to transiently express lower amounts of IL-33 receptor ST2, when compared with Th2 cells. However, IL-33 signaling can induce the expression of the lineage-specific transcription factors FOXP3, GATA-3, and T-bet for the positive activation of ST2 expression on Th1 cells (47). For regulatory T cells in the intestine, high level of ST2 are constitutively expressed and associated to the pathogenesis of eosinophilic pneumonia (48, 49).

In the mucosal barrier sites, IL-33 has been shown to coordinate the type 2 immune response through the activation of ST2-positive immune cells, such as ILC2s and CD4+ T cells (50). ILC2s are primarily localized at mucosal surfaces of lung, skin, gut and adipose tissues (51, 52) and play an important role in IL-33 associated allergic inflammatory diseases. Although ILC2s lack antigen receptors, they can be rapidly activated by the epithelial derived cytokines IL-33, IL-25, and thymic stromal lymphopoietin (TSLP), prostaglandin D2 from mast cells or cysteinyl leukotrienes secreted by activated hematopoietic cells (53–55). Activated ILC2 cells proliferate rapidly and act as an early innate source by producing large amount of the Th2 cytokines IL-5 and IL-13 in a synergistic manner (56). Bone marrow ILC2s has been shown to be a local source of IL-5 in IL-33-driven eosinophilia (57). Impaired Th2 cell differentiation was observed in ILC2 knockout mice (48), and the differentiation is in a cell-contact manner through major histocompatibility complex class II (49).

### Mast Cells and Basophils

Mast cells and its blood counterpart basophils, both play an important roles in allergic inflammation by generation and release of a panel of inflammatory mediators, such as histamine (58). Allergens can cross link with IgE sensitized mast cells to activate and release of large amounts of preformed and newly formed mediators: histamine, heparin, and proteases such as carboxypeptidase A3, chymase and tryptase (59–61). Most of these active proteases in the granule can cleave targets in nearby tissue compartments upon secreted from the activated mast cells (61). In human, mucosal mast cells express only tryptase and connective tissue mast cells express tryptase, chymase, and carboxypeptidase A3 (62). In mouse, mucosal mast cells express 2 chymase subtypes, mast cell protease (MCPT) 1 and MCPT2, whereas connective tissue type mast cells express the chymase MCPT4 and the elastase MCPT5, the tryptases MCPT6 and MCPT7, and carboxypeptidase A3 (62). IL-33 associated mast cell functions are involved in the pathogenesis of different allergic inflammations such as food anaphylaxis (63) and respiratory allergy induced by house dust mite or aspirin (54, 64, 65). Since the IL-33 receptor ST2 is constitutively expressed on mast cells, basophils and their progenitors cells, it is a critical amplifiers of IL-33–mediated allergic inflammation with the capacity in secreting a wide array of inflammatory cytokines and mediators (66). Antigen or IL-33 activated human mast cells can also release soluble ST2, which may further modulate the biologic effects of IL-33 (67). IL-33 has been demonstrated to enhance the adhesion of mast cells onto laminin, fibronectin, and vitronectin, increase the expression of adhesion molecules, such as ICAM-1 and vascular cell adhesion molecule-1 (VCAM-1) on endothelial cells, thus promoting mast cell adhesion to blood vessel walls (59). Mast cell survival, growth, development and maturation can be enhanced by IL-33 via the ST2/Myd88 pathway (68). Mast cell-derived tryptase and chymase have been demonstrated to cleave extracellular IL-33 into mature active forms (29) and IL-33 isoforms may have additional abilities to activate mast cells, thereby further provoking inflammation (69). Human mast cell chymase (HC) seems to be substrate specific. In a study using 51 active recombinant cytokines and chemokines (70), only 3 of them were substantially cleaved (IL-15 and two IL-1–related alarmins: IL-18 and IL-33) by HC.

The roles of mast cells proteases are not only associated with pro-inflammatory activities. In an *in vivo* study using ovalbumin (OVA)-sensitized mice lacking mouse MC protease 4 (mMCP4) (71), a chymase that is functionally equivalent to human chymase, the airway hyperresponsiveness when challenged with OVA was significantly higher in mMCP-4^(−/−)^ mice when compared with wild type mice. The thickness of the smooth muscle cell (SMC) layer was more pronounced in mMCP-4^(−/−)^ mice than in wild type control mice, thus indicating that chymase may have a modulating effect on airway SMCs. Taken together, the regulating role of chymase present in the upper airways could protect the animals against allergic airway responses. Therefore, the pro-inflammatory and anti-inflammatory effects of mast cells proteases may occur during different time frames, for example, initial activities that promote the airway response being followed by the mounting of protective activities that down-regulate the initial pro-inflammatory activities. Similar to mast cells, IL-33 mediates activation of human basophils and enhances their effector functions (58–60). Compared to mast cells, human basophils seem to have less amounts of proteases (61, 72). IL-33 also promotes asthma-related IL-4 and IL-13 production from basophils via MyD88-signaling pathway (73).

### Macrophages

**Figure 1:**
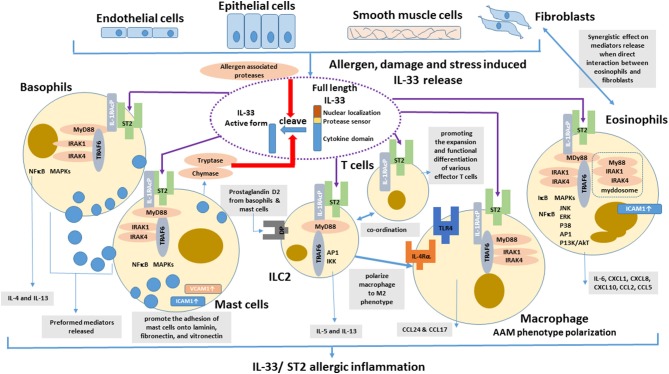
Effects of IL-33 on the activation of eosinophils, basophils, mast cells, DCs, ILC2 cells, and T cells in allergic inflammation. IL-33 is normally sequestered in the nucleus of various cells via nuclear-localization and chromatin-binding motifs in its amino terminus. After the cells are damaged, under stress, or stimulated by allergens, full-length IL-33 is released extracellularly, but it has low activity as a cytokine. Mast cells proteases and some allergens possess protease activity and can directly process IL-33 by cleaving within the protease-sensor domain to generate a more potent cytokine domain, which will directly activate local and infiltrating basophils, mast cells, group 2 innate lymphoid cells (ILC2), T cells, and eosinophils to induce allergic inflammation.

With abundant localization in lung tissue, macrophages are the important innate immune cells participating in allergic asthmatic inflammation (74). Th2 cytokines (IL-4 and IL-13) can polarize macrophage into alternative activated macrophage (AAM) phenotypes (75). Depletion of alveolar macrophages in murine acute allergic lung inflammation model demonstrated that Th2-immunity of allergic lung inflammation and airway remodeling were attenuated (76). IL-33 has been shown to promote the polarization of AAM that expressed mannose receptor and secreted CCL17 and CCL24 in an IL-13-dependent manner, thereby contributing to the airway inflammation in mice (77). IL-33 can enhance the lipopolysaccharide-mediated *in vitro* activation of macrophages, with the upregulation of the expression of toll-like receptor (TLR)4, myeloid differentiation protein 2, soluble CD14, and MyD88 (78). The updated effects of IL-33 on the activation of eosinophils, basophils, mast cells, macrophages, ILC2 cells and T cells in allergic inflammation is summarized in Figure 1.

### IL-33 in the Development of Allergy During Early Life

Recent murine studies have reported that there is a spontaneous accumulation of ILC2s, eosinophils, basophils and mast cells in the developing lung soon after birth, which is IL-33 dependent (79). Moreover, IL-33 is produced from type 2 airway epithelial cells (AEC2) during postnatal lung inflation (80). Large amount of IL-13 secreted from IL-33-activated ILC2 has been shown to polarize alveolar macrophages (AM) to anti-inflammatory M2 phenotype in newborn mice and contributed to lung quiescence in homeostasis with a delay in antibacterial effector responses for lifetime (80). On the other hand, exposure of allergen house dust mite during postnatal lung alveolarization further enhanced subsequent IL-33-induced Th2 cytokine production in activated ILC2s and CD11b+ dendritic cells (79, 81). Moreover, IL-33 inhibited IL-12 production and stimulated OX40L in neonatal dendritic cells, thereby promoting Th2 cell predominant for lung remodeling (79). House dust mites (HDM)-induced long-lasting Th2 immune response could be significantly neutralized by the intraperitoneal injection with recombinant soluble IL-33 decoy receptor in sensitization phase (79). This suppressive effect was even more significant in mice of young age than that of adult. Therefore, IL-33-ST2 axis is crucial for asthma development at childhood and intervention of such allergic axis is beneficial for the prevention of the later development of allergic asthma (79). Similarly, IL-33 concentration was found to be increased in the airways after exposure to *Staphylococcus aureus*–derived serine protease–like protein D (82).

### Recent Study of IL-33 and AD

Severe pruritus and skin inflammation are the main manifestations of poison ivy-induced allergic contact dermatitis (ACD). In a murine study, the central role of IL-33/ST2 signaling in pruritus and skin inflammation of this ACD has been illustrated (83), and the pruritic mechanism is associated with the interaction of IL-33/ST2 signaling with primary sensory neurons. Therefore, blockage of IL-33/ST2 signaling may represent a therapeutic target to relieve pruritus and skin inflammation of IL-33/ST2 signaling-related dermatitis (83). Apart from pruritic conditions, it has been shown that IL-33 are involved in boosting pain in a formalin-induced inflammatory pain mice model (84).

### IL-33 and Inflammatory Bowel Diseases (IBD)

Inflammatory bowel disease (IBD) is highly complex immune mediated sickness and mainly involved two disorders: Crohn's disease and ulcerative colitis with unclear pathophysiology. However, there are some clinical and pathophysiological similarities between IBD with asthma and non-pulmonary allergic diseases such as mast cell activity and the involvement of IgE (85). Moreover, upregulation of IL-33 and ST2 has been repeatedly demonstrated in the inflamed intestinal mucosa of IBD (86–90). Elevated IL-33 serum level of IBD patient has been shown to be reversed after anti-TNF-α treatment (87, 90). Different from asthma in which the AAM polarization is pro-inflammatory, IL-33 could prime macrophages into AAM in murine TNBS-induced colitis for inhibiting disease activity and the release of inflammatory mediators (91).

## IL-33 as The New Therapeutic Target for Allergic Diseases

Production of pro-inflammatory cytokines induced by IL-33 from ST2-expressing structural cells and hematopoietic cells including ILC2s, mast cells, Th2 cells, eosinophils, basophils, dendritic cells, and alternatively activated macrophages (AAM) is crucial to provoke atopic diseases such as allergic asthma and AD (83, 92, 93). *In vivo* murine studies with IL-33- and ST2-deficient transgenic mice, together with the analysis of patient samples further support the crucial role of the IL-33/ST2 axis in those allergic conditions (7, 33, 94, 95). Therefore, IL-33-blocking agents may be a novel therapeutic modality to treat allergic diseases and some promising compounds have recently been developed. Conventionally, glucocorticoids suppress the mRNA expression of pro-inflammatory mediators and exert broadly suppressive activities on inflammatory reactions via binding to the glucocorticoid receptors. IL-33-mediated pulmonary inflammation can be glucocorticoid resistant because other cytokines such as TSLP and IL-17 synergistically expressed at local inflammatory sites (50, 96). For example, allergic airway IL-33 production in house dust mite-induced murine asthmatic model was found to be corticosteroid-resistant (96). Compared with healthy controls, serum levels of IL-33 were significantly increased in psoriasis, psoriatic arthritis, and pustular psoriasis patients, and related to TNF-α. Anti-TNF-α therapy may also be effective against IL-33-related diseases (97). As the production of IL-33 is regulated by the upstream activation of ERK1/2, ERK1/2 inhibitors have also been shown to suppress the IL-33 production (98). The activation of β2-receptors and protein kinase A (PKA) could promote the IL-33 mRNA expression in dendritic cells, thereby suggesting that β-receptor blockers and PKA inhibitors may also be the candidates for IL-33–blocking agents (99, 100). Using mice model, butyrate has recently been found to inhibit proliferation and function of ILC2s by inhibiting intracellular GATA3 activity to suppress IL-33-mediated airway hyperresponsiveness and airway inflammation (101). Similar observations were found in human ILC2s, both *in vivo* and *in vitro* (101).

Proteases play important role in IL-33-mediated allergic diseases. Mast cell proteases are capable to cleave full length IL-33 to a more active IL-33 domain. It becomes a potential therapeutic target for IL-33 mediated allergic diseases. Endogenous protease inhibitors (cystatin A and SPINK5) have been shown to protect the airway epithelium from exogenous protease of patients with eosinophilic chronic rhinosinusitis (102). The development of protease inhibitor may exert therapeutic benefit in eosinophilic airway diseases.

The prolyl cis-trans isomerase proteinase inhibitor I (PIN1) is known to abnormally induce cytokines for eosinophil survival and activation by stabilizing cytokine mRNAs (103). Interleukin receptor associated kinase M (IRAK-M) is a PIN1 target critical for IL-33 signaling in allergic asthma (104). Nuclear magnetic resonance analysis with docking simulations suggests that PIN1 might regulate IRAK-M conformation and function in IL-33 signaling. The IL-33/ST2 signaling pathway recruits adapter protein MyD88 to transduce intracellular signaling (105, 106). MyD88 forms a complex with IL-R–associated kinases (IRAKs), IRAK4, and IRAK2, called the myddosome (MyD88–IRAK4–IRAK2). The myddosome subsequently activates downstream NF-kB, p38 MAPK, and JNK. A small synthetic molecule mimetics of α-helical domain of IRAK2 called compound 7004, which can inhibit the IL-33–induced NF-kB activity, disrupt myddosome formation, and attenuate the pro-inflammatory effects in an asthma-like animal model (105).

### Traditional Chinese Medicines (TCM) Targeting IL-33/ST2 Axis Against Allergic Inflammatory Diseases

Apart from the small molecules with specific target in the IL-33/ST2 axis as mentioned above, blocking IL-33 and its receptor by monoclonal antibodies is the major therapeutic approach in targeting IL-33/ST2 axis of allergic inflammatory diseases, and serval clinical trials are in progress (105, 107–111). The main side effect of monoclonal antibody administration is the risk of immune reactions such as serum sickness and acute anaphylaxis which may be fatal (112, 113). TCM and natural products may provide a great source of blockings agents against IL-33 activities. Some TCM formulae have been shown to be effective in attenuating IL-33 activities in both *in vitro* and *in vivo* studies (Table 1). Most of the component herbs in those formulae have been traditionally used to treat allergic and inflammatory diseases (39, 43, 127, 128).

**Table 1:** TCM formulae and natural compounds those are active against the IL-33/ST2 axis.

**Name**	**Uses, treatment or activities**	**Disease evaluated and animal model used/ human trials**	**Effect**	**References**
**Soshiho-tang:** Radix Bupleuri, Radix Scutellariae, Radix Ginseng, Tuber Pinelliae, Radix et Rhizoma Glycyrrhizae, Rhizoma zingiberis crudae, and Fructus Zizyphi	Pulmonary disorders such as the common cold and pneumonitis	Asthma,OVA-induced asthmatic mice model	Reduce leukocyte and eosinophilic counts, downregulate the production of IL-33 and other Th2-type cytokines, decrease mucus hypersecretion and IgE serum levels	(114, 115)
**Qingre-Qushi Recipe(QRQS):** Herba Hedyotis Diffusae, Radix Sophorae flavescentis, Herba Taraxaci, and Fructus Xanthii	Skin inflammation and itching	Atopic dermatitis (AD), OVA-induced atopic dermatitis mice model	Suppress both epidermal and dermal thickness, alleviating dermatitis and reducing IL-33 and ST2 positive cell numbers in OVA-induced AD mice Suppress the concentration of specific IgE, IgG, IgG1, and IgG2a antibodies in serum and the expression of IL-33, ST2, IL-1RAcP, IL-4, and IL-13 mRNA in the skin Down-regulate TNF-α and IFN-γ-induced IL-33 mRNA and protein expression in human keratinocyte HaCaT cells	(116)
**Calycosin**: A flavonoid, is a major component in Radix Astragli	Allergy related symptoms	AD, AD mice	Supress TSLP and IL-33 associated toll-liked receptor (TLR)4-mediated NF-κB signaling, the protein expression of MyD88, TIRAP, and TAK1	(117, 118)
**Cimifugin**: Bioactive and major component of Radix Saposhnikoviae	Allergy	AD, FITC sensitized, and challenged AD mice	Inhibit TSLP and IL-33 in the initial stage of AD to reduce the separated gap among the epithelial cells and increase the expression of tight junctions (TJs)	(119)
**Eupatilin**: Main lipophilic flavonoid obtained from the Artemisia species	Anti-oxidative, anti-inflammatory, and anti-apoptotic activities	AD, oxazolone-induced AD-like mouse model	An agonist of PPARα to ameliorate AD and restores the skin barrier function	(120–122)
**Protostemonine**: An alkaloid from Radix Stemonae	Anti-inflammatory activities	Asthma, DRA (dust mites, ragweed, and aspergillus)-induced murine asthma model	Inhibit pulmonary eosinophil infiltration, goblet cell hyperplasia, mucus secretion, IgE, and Th2 cytokine (IL-4, IL-5, IL-13, and IL-33) production Attenuate the expression of Arginase-1 (Arg-1), Ym-1, and Fizz-1, markers of AAM (alternatively activated macrophage) polarization,in lung tissues	(123)
**Tetramethoxyluteolin**: The structural analog of luteolin, a common flavonoids present in edible plants (e.g., carrots)	Suppressing mast cell activation	AD, Clinical trials	Inhibit mast cell activation stimulated by IL-33, substance P, or their combination Reduce skin inflammation in patients with AD in clinical studies	(124–126)

Besides TCM formulae, some natural compounds include flavonoids and alkaloids have been shown to be active against targeting IL-33/ST2 axis. Calycosin, a flavonoid, is a major component in Radix Astragli (117) that has been used in the treatment of allergy-related symptoms. When AD mice were treated with calycosin (0.4–10 mg/kg), the protein levels of TSLP and IL-33 were significantly suppressed (118). The inhibitory mechanism was associated to TLR4-mediated NF-κB signaling, with the significant inhibition of the expression of MyD88, toll/interleukin-1 receptor domain-containing adapter protein (TIRAP), and transforming growth factor beta-activated kinase 1 (TAK1) (118).

Cimifugin is a bioactive and major component of Radix Saposhnikoviae, a TCM has been used for treating allergy. Using FITC sensitized and challenged AD mice, cimifugin can significantly inhibit TSLP and IL-33 production in the initial stage of AD model. Moreover, cimifugin could reduce the separated gap among the epithelial cells and increase the expression of tight junctions (TJs). Similar effects on TSLP/IL-33 and TJs were obtained using keratinocyte HaCaT cells. Using siRNA blockage, cimifugin was found to inhibit initiative cytokines through restoring TJs. In addition, cimifugin administered alone in the initial stage obviously attenuated the ultimate allergic inflammation, thereby indicating the sufficient impact of cimifugin in the initial stage on TSLP/IL-33 and TJs for suppressing allergic inflammation. This study therefore implies the possibility of key cytokines such as IL-33 and TJs can be the therapeutic targets for AD (119).

Eupatilin (5,7-dihydroxy-30,40,6-trimethoxyflavone) is the major lipophilic flavonoid isolated from the Artemisia species (120). Eupatilin has been shown to promote the transcriptional activity and expression of peroxisome proliferator-activated receptor α (PPARα) in keratinocyte HaCaT cells (121) and acts as an agonist of PPARα to ameliorate atopic dermatitis (AD) and restore the skin barrier function. Eupatilin (20 ml of 1.5% or 3.0%) improved AD-like symptoms in an oxazolone-induced AD-like mouse model by suppressing the serum levels of IgE, IL-4, and AD-related cytokines including TNFα, IFN-γ, IL-1β, TSLP, IL-25, and IL-33 (122).

Protostemonine (PSN), an alkaloid isolated from Radix Stemonae was found to suppress inflammatory conditions, IL-33 production and polarization of macrophage into AAM phenotype in the lung tissues of a dust mites, ragweed and aspergillus-induced murine asthma model (123).

Tetramethoxyluteolin (methlut), a natural flavonoid, has been shown to inhibit mast cells stimulated by IL-33, substance P, or their combination. This has been further validated in a clinical trial in which a skin lotion containing tetramethoxyluteolin that can reduce skin inflammation in AD patients. In experimental study, methlut has also been shown to be effective in psoriasis conditions (124, 125).

Apart from Chinese herbs and natural products, acupuncture seems to be effective in attenuating the IL-33 associated airway inflammation in an OVA-induced mouse model by reducing the serum concentrations of IL-33, sST2, and other inflammatory cytokines (129). In summary, these TCM formulae and natural compounds could lower the IL-33 production and other inflammatory cytokines from the tested targets, thereby ameliorating the allergic symptoms. Some of them could target on the specific IL-33 associated immune cells type such as IL-33-mediated mast cell activation and macrophage polarization into AAM phenotypes (Table 1). In view of limited clinical evidence and laboratory studies on the action mechanism, further investigations on these two aspects are essential for the future development of TCM in IL-33-related diseases (126).

## Potential Future Developments and Concluding Remarks

With ample experimental evidences, the multiple roles of IL-33 in allergic and inflammatory diseases are not only restricted as an alarmin, but also as a cytokine for additional stimulatory signals: (i) to increase IL-33 expression in the nucleus or cytoplasm, and (ii) to induce IL-33 production into the extracellular space without cell death (29). Those stimulatory signals provide an amplification system for IL-33–mediated inflammatory responses. IL-33–blocking agents which target precisely at different molecular levels (both signaling and amplification pathways) could be potential therapeutic drugs for treatment of allergic and inflammatory diseases. For instance, an important mechanism for the direct activation of IL-33 by proteases from environmental allergens has been recently discovered (30). Targeting the “sensor” domain to prevent the cleavage and activation of IL-33FL, as well as the mast cell protease inhibitors might represent a new approach for reducing allergic responses in asthma and other allergic diseases. Apart from the pathological role in allergic diseases, IL-33 participates in diverse immune regulatory events. Therefore, to optimize the therapeutic outcomes, further evaluations, are essential to manipulate the IL-33/ST2 axis in diseases state and regulatory/physiological roles (130). As with other immunomodulating therapies, investigations on the effect of attenuating IL-33/ST2 axis on immune defense against infection and other immune responses are essential before further therapeutic development (131).

Since most of previous studies on IL-33 blocking agents are at the stage of *in vitro* and animal testing, pharmacological evaluations to develop IL-33–blocking agents are still on-going (132) and some are in phase I–II clinical trials for asthma and chronic obstructive pulmonary disease (133). The combination of IL-33 blocking agents may also be the synergistic intervention in IL-33-associated allergic and inflammatory diseases. For the future translational elucidation of IL-33, human studies are essential such as large scale clinical trials. Furthermore, the IL-33/ST2 axis is participating in both Th2/IL-31 and Th17 immune response during the progression of allergic airway diseases (92). Natural products and herbal medicines with the pluripotent activities to inhibit the production and actions of IL-33 are also promising candidates for further pharmacological evaluation for the treatment of allergic diseases. TCM and natural products, especially flavonoids with proven *in vitro* and *in vivo* activities to target the IL-33/ST2 axis, are potential candidates and warrant further development for the lead compounds as adjuvant anti-allergic and anti-inflammatory agents.

## Author Contributions

CW and BC wrote the manuscript and prepared the figure. L-ST, CL, and CW drafted sections, structured, and edited the manuscript.

### Conflict of Interest Statement

The authors declare that the research was conducted in the absence of any commercial or financial relationships that could be construed as a potential conflict of interest.
